# A Case of Acute Aortic Dissection in an Older Patient of Advanced Age

**DOI:** 10.7759/cureus.74567

**Published:** 2024-11-27

**Authors:** Tomohiro Nakajima, Kei Mukawa, Yutaka Iba, Tsuyoshi Shibata, Nobuyoshi Kawaharada

**Affiliations:** 1 Cardiovascular Surgery, Sapporo Medical University, Sapporo, JPN

**Keywords:** acute aortic dissection, atrial fibrillation, cerebral infarction, stroke, super-elderly

## Abstract

The patient an 85-year-old female resided in a care facility where she maintained an independent daily activity level. She was discovered hunched over a table in her room, displaying reduced responsiveness and prompting an emergency call. Initially, her blood pressure was within 60 mmHg, and she was transported by ambulance to our hospital. Further examination revealed acute Stanford type A aortic dissection accompanied by signs of cardiac tamponade, necessitating urgent surgery.

The operation was performed under general anesthesia and tracheal intubation. After exposing the femoral vessels through an incision in the right groin, cannulation was achieved for cardiopulmonary bypass. Subsequently, a median sternotomy was performed and the pericardium was opened. Blood within the pericardial cavity was carefully exposed and blood pressure was monitored. The pericardial cavity contained a large number of dark red hematomas.

A left ventricular vent was inserted and cooling was initiated. The circulatory arrest was achieved at a rectal temperature of 28°C, accompanied by antegrade cerebral perfusion and selective antegrade myocardial protection to facilitate cardiac arrest. The entry tear was located on the dorsal aspect of the ascending aorta. Additionally, the ascending aorta was trimmed proximal to the brachiocephalic artery and a 26-mm Gelweave graft was anastomosed. Circulation was subsequently resumed, and rewarming commenced.

The proximal dissection was extended to the non-coronary cusp, where BioGlue was applied to bond the intima and adventitia, followed by a partial adventitial inversion. The proximal anastomosis was then completed. The total operation duration was 366 min. The patient was extubated, and oral intake was initiated the following day. However, postoperative delirium persisted, and the patient developed a cerebral infarction triggered by paroxysmal atrial fibrillation. Her daily activities declined, and she experienced complications including pneumonia and urinary tract infection, which responded to antibiotic therapy. The patient was discharged on postoperative day 49.

## Introduction

With an aging population, the incidence of aortic dissection in older patients, particularly those over 80 years of age, is on the rise [1]. Aortic dissection, which is characterized by a tear in the aortic wall, is a life-threatening cardiovascular disease with significant morbidity and mortality. Older patients often exhibit a high prevalence of risk factors for aortic dissection, including hypertension, arteriosclerosis, and other cardiovascular conditions, which further complicates disease management. Despite advancements in surgical techniques, the management of aortic dissection in patients aged > 80 years presents unique challenges. Older patients often have comorbidities such as renal dysfunction, diabetes, and respiratory diseases, which elevates the risks associated with surgical intervention. These comorbidities can aggravate postoperative outcomes and raise concerns regarding patient safety and quality of life [2].

Furthermore, while surgery remains a critical treatment for aortic dissection, older patients experience an elevated perioperative risk. Studies have demonstrated high rates of postoperative complications in older patients, including renal failure, stroke, and infections. This case report describes an 85-year-old patient who underwent emergency surgery for acute aortic dissection [3]. Postoperatively, the patient experienced an ischemic stroke due to paroxysmal atrial fibrillation, leading to decreased activities of daily living (ADL). This report discusses the challenges in managing aortic surgery in older patients, particularly regarding postoperative risks and complications [4].

## Case presentation

**Figure 1:**
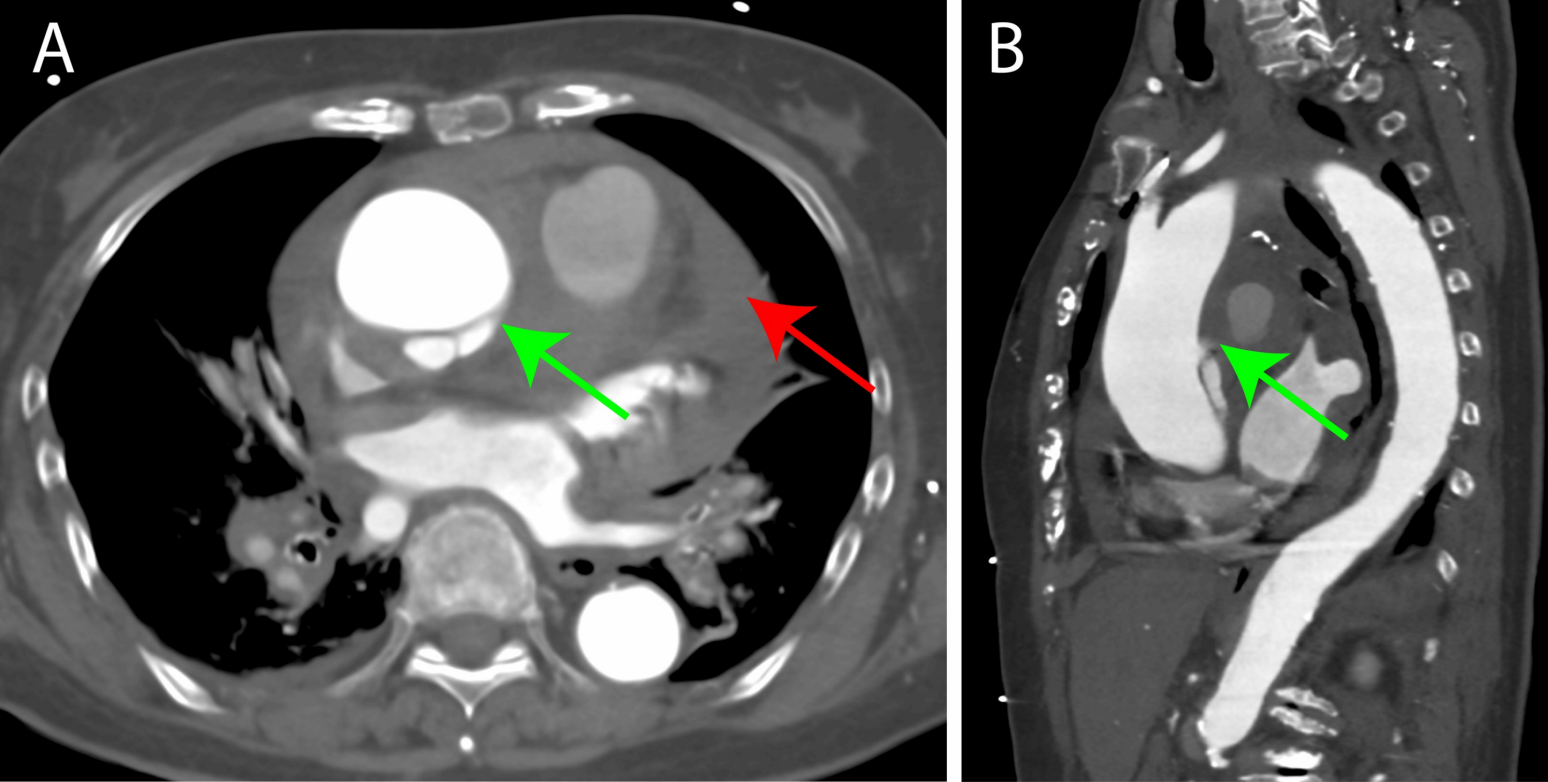
Preoperative computed tomography findings (A) The horizontal section displays an entry of dissection in the dorsal aspect of the ascending aorta (green arrow) and an intrapericardial hematoma (red arrow). (B) A sagittal section demonstrating dissection entry on the dorsal aspect of the ascending aorta (green arrow).

An 85-year-old female, residing in a care facility maintained independence in daily living with a Clinical Frailty Score of 2. On the morning of admission, she was discovered by staff delivering breakfast slumped over a table in her room. Unresponsive and hypotensive, with a blood pressure of 60 mmHg. Therefore, she was transported to our hospital by ambulance. Echocardiography revealed a pericardial effusion. Contrast-enhanced computed tomography (CT) confirmed the diagnosis of acute Stanford Type A aortic dissection (ATAAD) (Figure 1). Consequently, an emergency operation was performed.

**Figure 2:**
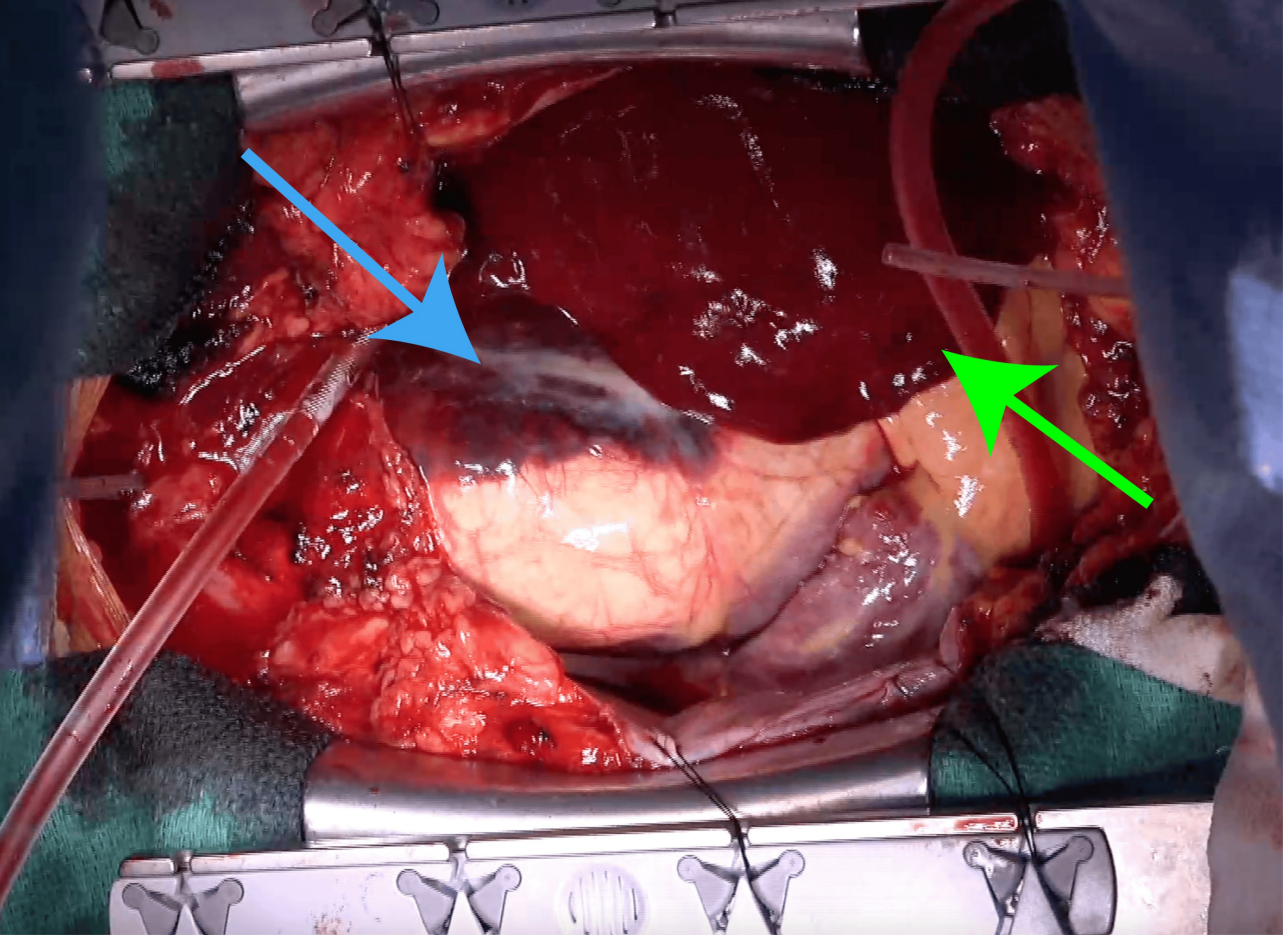
Operative image An intrapericardial hematoma is observed (green arrow). A hematoma is observed extending from the ascending aorta toward the pulmonary artery (blue arrow).

Under general anesthesia, the right groin was incised to expose the femoral vessels, and cannulas for cardiopulmonary bypass (CPB) were placed. A median sternotomy was performed, and the pericardium was opened, revealing a large volume of dark red hematomas. Subsequently, the hematomas were evacuated (Figure 2). A left ventricular vent was inserted and systemic cooling was initiated. The circulatory arrest was achieved at a rectal temperature of 28°C.

**Figure 3:**
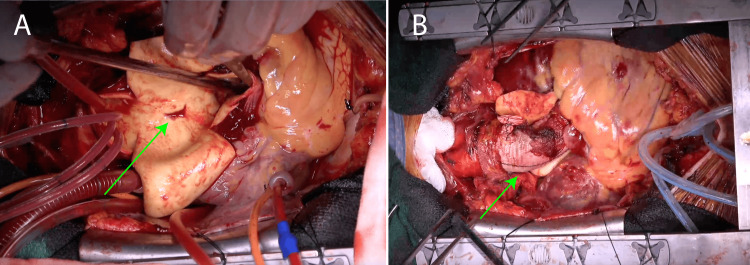
Intraoperative images (A) Entry is observed in the dorsal aspect of the ascending aorta (green arrow), (B) The ascending aorta is replaced with an artificial vessel (green arrow).

The ascending aorta was incised and the entry site on the dorsal aspect was identified (Figure 3A). Hematoma formation extended toward the pulmonary artery. Antegrade cerebral perfusion and selective antegrade cardioplegia were initiated and cardiac arrest was sustained with retrograde coronary perfusion every 25 min. The ascending aorta was trimmed proximal to the brachiocephalic artery and replaced with a 26-mm Gelweave graft (Vascutek Terumo Inc., Scotland, UK). The dissection extended to the non-coronary cusp, necessitating reinforcement of the aortic wall with BioGlue (CryoLife International Inc., Kennesaw, GA, USA) and adventitial inversion before proximal graft anastomosis (Figure 3B).

**Figure 4:**
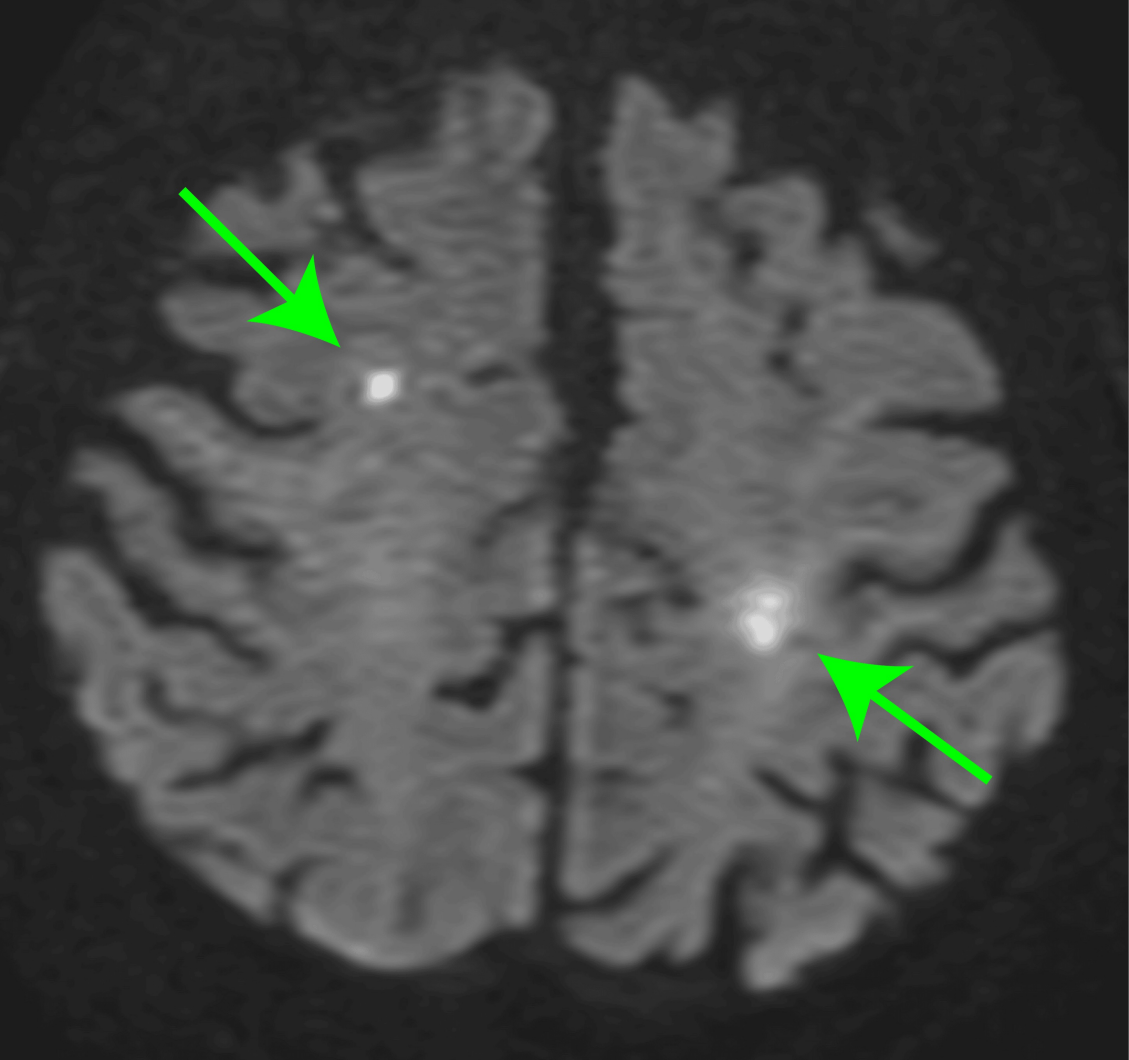
Magnetic resonance imaging of the head Diffuse cerebral infarct lesions in the bilateral cerebrum (green arrows).

The patient was rewarmed and weaned from the CPB, and the chest was closed. The total surgical time was 366 min. The patient was extubated the following day, and oral intake was initiated. However, delirium persisted, and on postoperative day 5, the patient developed an ischemic stroke secondary to paroxysmal atrial fibrillation (Figure 4). Her ADL deteriorated; she was initially able to ambulate with a walker but eventually became predominantly bedridden. Deconditioning led to complications, such as pneumonia and urinary tract infection, which were resolved with the administration of cefepime. The patient was transferred to a rehabilitation facility on postoperative day 49, with a Clinical Frailty Score of 7.

## Discussion

The management of ATAAD in older patients, particularly those aged 80 years and above, presents unique challenges [5]. Despite advances in surgical techniques and perioperative care, older adults who undergo complex surgeries often experience significant complications that affect their survival and recovery. In this report, we discussed the challenges encountered during the postoperative management of an 85-year-old patient, emphasizing frailty, comorbidities, and the risk of adverse outcomes [6].

Older patients are likely to present with frailty and multiple comorbid conditions including chronic hypertension, diabetes, and impaired renal function, all of which impact surgical outcomes. Frailty, defined as a reduced physiological reserve and resistance to stressors, is a critical determinant of postoperative outcomes. This patient, with a preoperative Clinical Frailty Score of 2, developed worsening frailty postoperatively (score of 7), reflecting profound physical deconditioning and the cumulative burden of complications including pneumonia, urinary tract infection, and ischemic stroke [7]. Studies suggest that frailty in older patients with ATAAD correlates with prolonged hospital stays and reduced quality of life, underscoring the need for tailored preoperative assessments. Postoperative care complications, such as delirium and stroke, further complicate the recovery process. Stroke, particularly, remains a frequent and devastating complication of ATAAD surgery, with a reported incidence of 10-20% in older patients. The patient developed an ischemic stroke secondary to paroxysmal atrial fibrillation, resulting in a significant functional decline. Proactive strategies including close cardiac rhythm monitoring and early anticoagulation protocols may mitigate these risks. However, these interventions must be balanced against the heightened risk of bleeding inherent in this population [8].

Furthermore, the response and healing capacity of older adults is often impaired, contributing to increased susceptibility to infections such as pneumonia and urinary tract infections. These conditions prolong hospital stays, delay rehabilitation, and increase mortality risk. Optimized infection prevention protocols, early mobilization, and targeted rehabilitation programs are critical for improving outcomes.

In conclusion, although intervention remains the standard of care for ATAAD, its application in older patients requires meticulous perioperative planning and postoperative care. Frailty, cognitive dysfunction, and infections are key contributors to adverse outcomes and require an interdisciplinary approach to management.

## Conclusions

The management of ATAAD in older patients is fraught with difficulties owing to their heightened frailty, comorbidities, and vulnerability to postoperative complications. Despite the successful surgical intervention, the patient experienced significant functional decline and postoperative complications, including stroke and infection. These outcomes underscore the importance of frailty assessment and tailored perioperative care in this high-risk group.
